# Higher Prevalence of Chronic Endometritis in Women with Cesarean Scar Defect: A Retrospective Study Using Propensity Score Matching

**DOI:** 10.3390/jpm13010039

**Published:** 2022-12-24

**Authors:** Longlong Wei, Chunyu Xu, Yan Zhao, Cuilian Zhang

**Affiliations:** 1 Zhengzhou University People’s Hospital, Zhengzhou 450052, China; 2 Department of Reproductive Medicine Center, Henan Provincial People’s Hospital, Zhengzhou 450003, China

**Keywords:** cesarean section, cesarean scar defect, chronic endometritis, propensity score matching, infertility

## Abstract

(1) Background: A cesarean scar defect may cause localized inflammation of the endometrial tissue, and various researchers believe that the presence of a cesarean scar defect is associated with chronic endometritis. However, there is no report on the possible association between cesarean scar defects and chronic endometritis thus far. This study aimed to assess the role of having a cesarean scar defect in a person’s susceptibility to chronic endometritis. (2) Methods: This retrospective propensity-score-matched study comprised 1411 patients with chronic endometritis that were admitted to Henan Provincial People’s Hospital in China from 2020 to 2022. Based on whether a cesarean scar defect was present or not, all cases were assigned to the cesarean scar defect group or the control group. (3) Results: Of the 1411 patients, 331 patients with a cesarean scar defect were matched to 170 controls. All unbalanced covariates between groups were balanced after matching. Before matching, the prevalence of chronic endometritis in the cesarean scar defect group and in the control group was 28.8% and 19.6%, respectively. After correcting for all confounding factors, a logistic regression analysis showed that cesarean scar defect occurrence may increase the risk of chronic endometritis (odds ratio (OR), 1.766; 95% confidence interval (CI), 1.217–2.563; *p* = 0.003). After matching, the prevalence of chronic endometritis was 28.8% in the cesarean scar defect group and 20.5% in the control group. Thus, even after correcting for all confounding factors, the logistic regression analysis still showed that a cesarean scar defect remained an independent risk factor for chronic endometritis prevalence (OR, 1.571; 95% CI, 1.021–2.418; *p* = 0.040). The findings were consistent throughout the sensitivity analyses. (4) Conclusions: The present results suggest that the onset of a cesarean scar defect may increase the risk of chronic endometritis.

## 1. Introduction

In the literature, the poor healing of local incisions after a cesarean section (CS) is termed a cesarean scar defect (CSD) [1,2], isthmocele [3], diverticulum [4], etc., with the most common term being CSD. In this study, a CSD is defined as a depression or cavity that connects with the uterine cavity because of the poor healing of the local uterine incision after receiving a CS [5,6,7]. Patients with a CSD experience uterine cavity effusion, which appears as menostaxis, due to the persistently inadequate drainage of menstrual blood [8]. Additionally, the persistent uterine effusion may further cause localized inflammation of the endometrial tissue [9]. Chronic endometritis (CE) is described as a localized edematous inflammation of the uterine mucosa [10], which can affect endometrial receptivity, implantation, and pregnancy outcomes [11,12,13,14,15].

Various researchers believe that the presence of a CSD is associated with CE. Morris et al. investigated the pathological changes in the CSD area in 51 hysterectomy cases, in which patients presented moderate to marked lymphocytic infiltration [16]. A study by Higuchi A studied 63 cases of patients who underwent CSD lesion laparoscopic resection, and suggested that the expression of a chronic inflammatory marker was higher in women with a CSD [17]. These findings above suggested the presence of a chronic inflammatory marker in the CSD area. A higher prevalence of CE in women with a CSD was also anticipated. However, there was a lack of evidence regarding this speculation.

The prevalence of CSDs is low, and their causes are complex. Simultaneously, CSDs are difficult to artificially control and randomly assign. Thus, a randomized controlled trial regarding CSDs may be difficult to achieve and may result in serious ethical violations. Propensity score matching (PSM), which may eliminate selection bias in observational studies and the uneven distribution of prognostic factors between groups, has proven to be a novel, practical, and creative statistical approach to assess the effects of interventions in nonrandomized controlled data [18]. This study aimed to evaluate the role of CSDs in a person’s susceptibility to CE using PSM.

## 2. Methods and Materials

### 2.1. Recruitment of Patients

This case–control study comprised candidates for hysteroscopy due to infertility who attended the Reproductive Medicine Center of Henan Provincial People’s Hospital between 2020 and 2022. According to the inclusion and exclusion criteria, all of the patients who participated were carefully chosen. In our center, the indications for hysteroscopy are as follows [19,20,21,22,23,24,25]: (1) a routine physical examination before the application of assisted reproductive technology; (2) abnormal uterine bleeding; (3) an ultrasound or imaging examination such as hysterosalpingography that suggests abnormal echogenicity of the uterine cavity; and (4) women with repeated implantation failure, recurrent miscarriage, etc. All study procedures were reviewed and approved by the ethics review board of the hospital (approval number: SYSZ-LL-2021091501).

The inclusion criteria were: (1) secondary infertility after a CS or natural vaginal birth and (2) the presence of informed consent. The exclusion criteria were as follows: (1) uterine abnormalities, including malformation, submucosal fibroids, endometrial polyps, or uterine adhesions; (2) recurrent miscarriage (≥3 consecutive miscarriages before gestational week 20); (3) acute pelvic inflammation, cervicitis, or vaginitis; (4) a history of tuberculosis infection; (5) hydrosalpinx; (6) receipt of therapeutic medicines that may affect the survival status of bacteria in the past month, including antibiotics, glucocorticoids, or immunosuppressants; (7) women who were previously diagnosed with CE; and (8) a diagnosis of endometriosis.

### 2.2. Definitions

The medical records included demographic features and gynecologic and medical history, which were extracted as patient characteristics. A prolonged menstrual period was defined as a period lasting over 7 days [26,27]. If a patient was pregnant or gave birth during this study, the gravidity and parity at the time of hysteroscopy was used as data for our records. If a patient underwent more than one hysteroscopy, only the first procedure was examined.

Hysteroscopy combined with transvaginal ultrasound was used to diagnose the CSD [8,28,29,30]. Failure to conceive after one year of regular intercourse without contraception was defined as infertility [31]. Hydrosalpinx was diagnosed via a transvaginal ultrasound scan [32]. Uterine adhesions were diagnosed via hysteroscopy [33]. Hysteroscopy combined with three-dimensional ultrasound was used to diagnose uterine abnormalities [34,35]. Endometriosis was diagnosed via ultrasound and laparoscopy [36].

As mentioned in Section 2.3, the diagnostic criteria for CE in other studies was controversial. Hence, different diagnostic criteria were selected for our statistical analysis. The alternative criteria are: a diagnosis of CE when hysteroscopic-positive features accompanied by a plasma cell count ≥ 5/high-power field (HPF) were detected through endometrial biopsy.

### 2.3. Diagnosis of CE

The presence of plasma cells in endometrial tissue is the generally accepted gold standard for the diagnosis of CE. However, conventional HE staining has the disadvantage of difficulty in identifying plasma cells and is highly dependent on the experience of pathologists. Accordingly, transmembrane acetyl heparan sulfate proteoglycan (CD138) has recently been used as a particular marker of plasma cells to aid in the diagnosis of CE [37]. Despite the widespread use of CD138+ plasma cell detection for the diagnosis of CE, no agreement on the minimum number of positive plasma cells required to define CE has been reached [38].

Reviewing the previous literature, we determined that the following diagnostic criteria were used in previous studies [39,40,41,42]: (1) more than one plasma cell/HPF; (3) more than five plasma cells/HPF; (4) more than one plasma cell per section; (5) more than one plasma cell per 10 HPFs; and (6) an endometrial stromal plasmacyte density index ≥ 0.25. Moreover, the diagnosis of CE based on pathology alone is, possibly, controversial [43]: (1) the minimal amount of tissue obtained when performing the endometrial biopsy may cause possible false negatives; (2) a healthy, fertile woman’s endometrium could also have few endometrial stromal plasma cells; (3) contamination from basal uterine tissue or cervical canal tissue may cause possible false positives. Hysteroscopy has been shown to increase the sensitivity/accuracy of a CE diagnosis.

In order to increase the precision of the CD-138 immunohistochemistry, hysteroscopy can be a helpful screening method for chronic endometritis. According to recent data, hysteroscopy might have been a helpful add-on technique for narrowing the “gray area” of those cases classified with mild CE conditions (<5 plasma cells/HPF) [44]. Additionally, it was found that individuals undergoing IVF cycles had a greater predictive value for a CE diagnosis via hysteroscopy compared to CD-138 immunohistochemistry [45]. Therefore, various scholars advocate for the use of hysteroscopy combined with histopathology to improve diagnostic efficiency.

Taking into account the literature, the actual work of our center, and the opinions of some experts in the field, CE was defined in this study according to the endometrial biopsy and hysteroscopy findings.

#### 2.3.1. Hysteroscopy and Sample Collection

**Figure 1:**
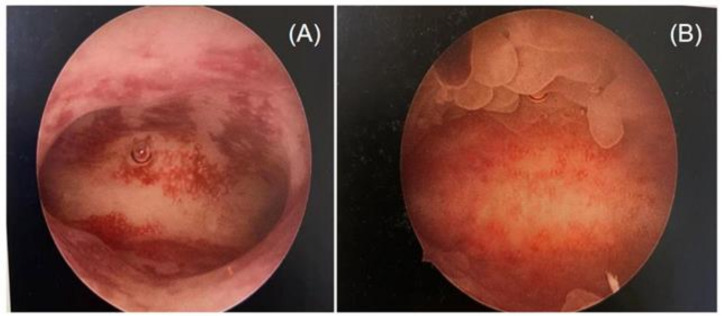
Different features of chronic endometritis at hysteroscopy: (**A**) diffuse hyperemia endometrium; (**B**) micropolyps (less than 1 mm in size).

Two highly skilled hysteroscopists specializing in infertility and reproductive endocrinology performed the hysteroscopy simultaneously. Since performing an endometrial biopsy during different menstrual cycles has been proven to have an impact on the diagnosis of CE [37,46], all operations were conducted during the follicular phase of the menstrual cycle in this study. Photographs of the hysteroscopic procedures were obtained and recorded in the working platform’s digital format. Stromal edema, diffuse or localized hyperemia, “strawberry aspect” (hyperemic patches interrupted by white or pale focal areas), and micropolyposis (endometrial polyps less than 0.1 cm) were all judged to be indicators of CE through the hysteroscopic examination [25,47] (Figure 1).

#### 2.3.2. Histological and Immunochemistry Analysis

When a positive finding was detected through hysteroscopy, all parts of the uterine cavity were biopsied and a sample was placed in formalin for pathology. Hematoxylin and eosin was used to stain tissue samples. Additionally, CD138-positive plasma cells were identified using immunohistochemistry (IHC) labeling after being incubated with a mouse monoclonal antibody against human CD138 (diluted 1: 100, DAKO, Denmark) overnight at 4 °C [48,49,50].

When interpreting the results of the endometrial biopsy, the presence of one plasma cell per HPF was indicated as a positive result.

### 2.4. Propensity Score Matching (PSM)

To estimate the causal effect of a CSD on the prevalence of endometritis, we used PSM to mimic a randomized controlled trial. The baseline covariates selected for PSM were based on high risk factors for CE identified in the literature. The final covariates were age, body mass index (BMI), gravidity, parity, infertility duration, history of intrauterine device (IUD) use, and menstruation situation. Propensity score values were estimated via a logistic regression analysis and matched using the 1:2 nearest neighbor method. The process ensured the goodness of the matching results by defining the caliper value, which was set at 0.2. Subsequently, the change in covariates between groups before and after matching was compared, and the closer the standard difference after matching was to 0, the more satisfactory the matching results were. The balance of variables between groups was considered good when the absolute value of the standard difference was <0.1 (10%).

### 2.5. Statistics

Continuous data were presented as $\bar{X}$ ± s or M (P25, P75), as applicable, whereas categorical data were given if in proportions. Depending on the distribution pattern, Student’s *t*-test or a nonparametric Mann–Whitney U test was employed to determine if each dataset had a normal distribution when using the Kolmogorov–Smirnov test. Qualitative variables were compared by using the chi-square and Fisher exact tests. Balance at the baseline in both groups was evaluated using the standardized mean difference (SMD). All confidence intervals (CI) were 95%. Statistical significance was defined as a *p*-value < 0.05. All data were managed and analyzed using SPSS version 23.0 and R’s programming environment (Version 4.2.1.). A sensitivity analysis was performed by discussing the controversy of the diagnostic criteria for CE. As mentioned in Section 2.3, the diagnostic criteria for CE used in other studies were controversial. Different diagnostic criteria were selected for the statistical analysis to eliminate any bias.

## 3. Results

### 3.1. Baseline Characteristics before and after Propensity Score Matching

**Figure 2:**
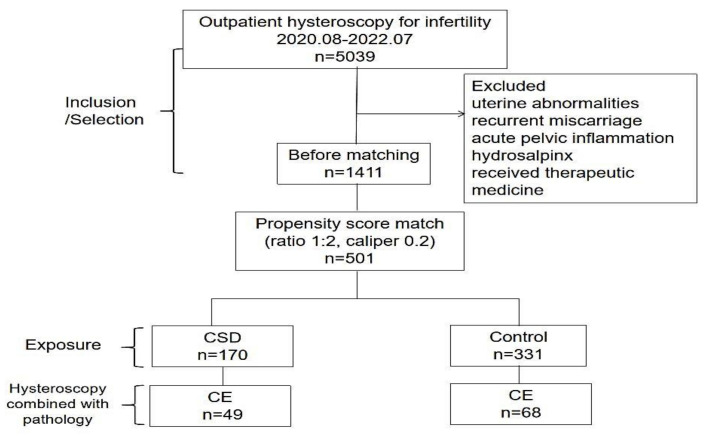
Study flow-chart. Note: CSD–cesarean scar defect; CE–chronic endometritis.

After applying the exclusion criteria, 1411 patients were included. Of all included patients, 170 cases of CSDs were matched (1:2) to 331 control cases, and some cases were discarded due to difficulties in meeting the matching criteria (Figure 2).

**Figure 3:**
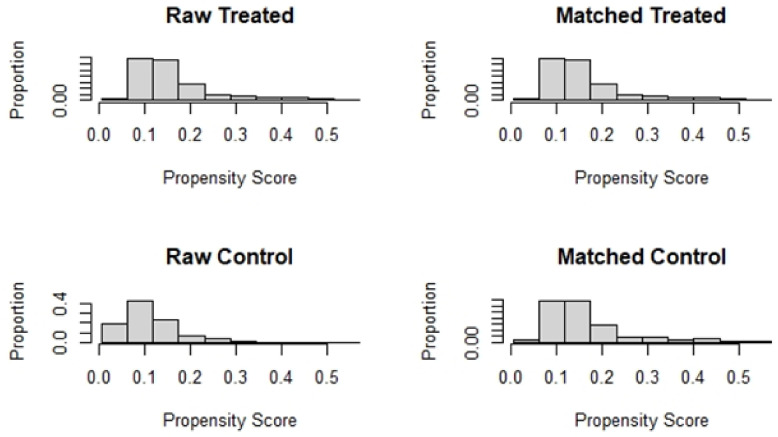
Histogram of data distribution before and after matching.

Table 1 compares the baseline characteristics before and after matching. The absolute values of the SMD for each index before matching ranged from 0.0240–0.5804. The absolute values of the SMD after matching were all controlled within 10%. All baseline variables were more balanced after PSM (Figure 3).

### 3.2. Outcomes before and after Matching

**Figure 4:**
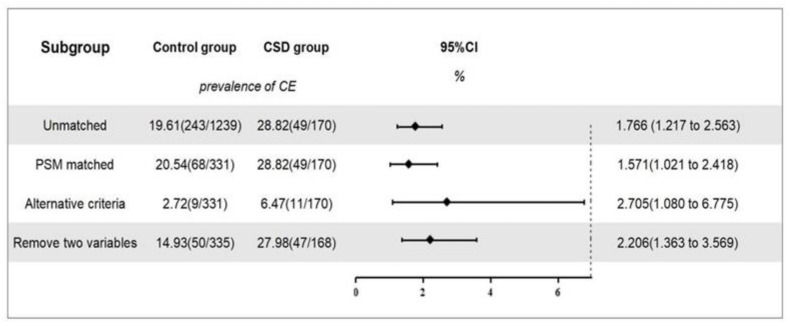
The prevalence of CE and sensitivity analyses. Note: Overview of the association between CSD and the prevalence of CE and sensitivity analysis. Alternative criteria: CE was diagnosed when a hysteroscopic-positive feature was present and accompanied by plasma cell count ≥5/HPF in endometrial biopsy. Remove two variables: age and BMI were removed as propensity score matching variables, and the logistic regression analysis was conducted again.

Before matching, there were 1239 women in the control group, of which 243 were diagnosed with CE. The overall prevalence was 19.6%. There were 170 cases in the CSD group, of which 49 were diagnosed with CE, with a prevalence of 28.8%. After correcting for all confounding factors, including age and BMI, a logistic regression analysis showed that a CSD was associated with the occurrence of CE (OR, 1.766; 95% CI, 1.217–2.563; *p* = 0.003; Figure 4).

After matching, there were 331 cases in the control group, of which 68 were diagnosed with CE, with a prevalence of 20.5%. There were 170 cases in the CSD group, of which 49 were diagnosed with CE, with a prevalence of 28.8%. After correcting for all confounding factors, including age and BMI, a logistic regression analysis showed that a CSD was significantly associated with the occurrence of CE (OR, 1.571; 95% CI, 1.021–2.418; *p* = 0.040; Figure 4).

CSD was associated with a susceptibility to CE before (OR, 1.766; 95% CI, 1.217–2.563; *p* = 0.003) and after (OR, 1.571; 95% CI, 1.021–2.418; *p* = 0.040) PSM. These estimations remained stable across all sensitivity analyses (Figure 4).

In a second sensitivity analysis (*n* = 503), after removing age and BMI as PSM variables, the association between CSD and the prevalence of CE (OR, 2.206; 95% CI, 1.363–3.569; *p* = 0.001) remained relevant (Figure 4).

## 4. Discussion

This large case–control study regarding the relationship between CSDs and CE can provide reliable evidence for clinical decisions. In this study, we used propensity score matching to match the data of infertile women with and without a CSD, and a total of 501 cases were matched successfully. A total of 28.8% of patients with a CSD were diagnosed with CE, which is a relatively high frequency. Moreover, the frequency of CE in the control group was 20.54%. Generally, the prevalence of CE ranges from 8% to 72% [51]. We assumed that this may be related to the patients selected. All the women involved in this study were infertile. Only one difference existed among the successfully matched patients (whether they had a CSD), and the differences in all other covariates were eliminated. It can be assumed that these women were randomized into the CSD and control groups. After correcting for all confounding factors, a logistic regression analysis still showed that a CSD remained an independent risk factor for CE (OR, 1.571; 95% CI, 1.021–2.418; *p* = 0.040). Additionally, these findings were consistent throughout the sensitivity analyses, which increases the credibility of this study.

A CSD, described for the first time as an “isthmocele” by Morris in 1995, was considered to be a risk factor for inflammation in the uterine cavity. In a study by Morris et al., pathological changes in the CSD were reported for a series of 51 hysterectomy cases. The changes involved moderate to marked lymphocytic infiltration and capillary dilatation [16]. Higuchi A et al. also reported that some chronic inflammatory markers, such as CD138, were observed in the CSD area [17]. These findings suggest the presence of chronic inflammation in the CSD area. We assumed that chronic inflammation in the CSD area may cause further changes in the uterine environment, as they are interconnected. A study by Yang et al. analyzed 16S recombinant DNA (rDNA) of endometrium flora in the CSD population and noted that there was a similar pattern in the interrupted microbial flora at each level in infertile women that received a CS. Moreover, compared with the controls, there was a lower lactobacillus-dominating percentage in the CS group. This change could be a sign of chronic endometritis [11]. Our findings further confirmed this suspicion.

Researchers have reported a lower pregnancy rate in patients who previously underwent a CS delivery compared with patients who previously delivered vaginally after ART [52]. Although the CSD area is located at the cervical uterine junction, this is far away from where we would assume the implantation to occur. A high prevalence of CE in women with a CSD may be one of the causes of this situation. Although patients with CE may not have any clinical symptoms, there has been much research conducted that shows the relationship between CE, infertility, and implantation failure [38,42,53,54,55].

A large amount of research suggests that standardized antibiotics are effective at clearing CE and improving the reproductive prognosis of patients [56]. This study suggests that conducting a hysteroscopy and endometrial biopsy in infertile women with a CSD as early as possible might be beneficial. Further studies are needed regarding this hypothesis.

This study has some limitations. First, this study applied propensity score matching to balance the differences between the CSD and control groups. However, this method can only balance observable variables and can do nothing about the bias caused by potential unknown confounding variables. Second, a history of vaginitis, gravidity, and parity was recorded based on a patient’s statement, but this may not be reliable due to recall bias. Third, this study is a single-center study; therefore, the results of thehysteroscopy, endometrial biopsy, and pathology may be heterogeneous as compared with other centers. Fourth, because of the invasiveness and high price of histological and immunochemistry analyses, we performed histological and immunochemistry analyses only in women with features of endometritis through hysteroscopy. This may have omitted the data of some patients with CE. The reason for not having included a similar number of subjects and controls was that the low prevalence of CSDs resulted in a small number of control groups. To eliminate the bias and increase the credibility of this study, a 1:2 propensity score match was chosen to maximize the sample size. However, this is not a limitation.

## 5. Conclusions

Our study evaluated the hypothesis that the onset of a CSD may increase the risk of CE. We concluded that conducting a hysteroscopy and endometrial biopsy in infertile women with a CSD as early as possible might be beneficial. Further studies are needed regarding this hypothesis.

## Figures and Tables

**Table 1:** Baseline characteristics before and after propensity score matching.

Baseline	Control Group Prematch (*n* = 1239)	Control Group Postmatch (*n* = 331)	CSD Group (*n* = 170)	Standard Difference Prematch	Standard Difference Postmatch
Age (y, $\bar{X}$ ± s)	32.50 ± 3.52	34.98 ± 4.99	34.95 ± 4.31	0.5804	−0.0529
BMI (kg/m^2^, $\bar{X}$ ± s)	23.5 ± 3.52	23.73 ± 3.72	23.69 ± 3.61	0.0240	−0.0177
Gravidity (*M*, *P_25_*, *P_75_*)	2.89 (1.0, 4.0)	2.53 (1.0, 3.0)	2.42 (1.0, 3.0)	−0.3421	−0.0611
Parity (*M*, *P_25_*, *P_75_*)	1.27 (1.0, 2.0)	1.13 (1.0, 1.0)	1.16 (1.0, 1.0)	−0.2706	0.0881
Infertility duration (y, $\bar{X}$ ± s)	3.73 ± 2.62	3.80 ± 2.79	3.79 ± 2.41	0.0228	−0.0090
History of IUD (*n* (%))					
Yes	22 (1.8)	9 (2.7)	5 (2.9)	0.0673	0.0175
No	1217 (98.2)	322 (97.3)	165 (97.1)	0.0498	0.0267
History of prolonged menstruation (*n* (%))					
Yes	50 (4.0)	20 (6.0)	17 (10.0)	0.2237	0.0844
No	1189 (96.0)	311 (94.0)	153 (90.0)	0.3564	0.0767

Note: CSD–cesarean scar defect; BMI–body mass index.

## Data Availability

Not applicable.
